# Jingdezhen ceramic culture in the digital era: a qualitative inquiry into digital dissemination and platform innovation

**DOI:** 10.3389/fdata.2026.1752142

**Published:** 2026-03-09

**Authors:** Qiuyang Huang, Zhengjun Chen

**Affiliations:** School of Modern Services, Jiangxi Arts and Ceramics Technology Institute, Jingdezhen, Jiangxi, China

**Keywords:** cultural identity, cultural innovation, digital dissemination, Jingdezhen ceramics, livestreaming commerce, platform economy, qualitative research

## Abstract

**Introduction:**

Digital platforms have increasingly reshaped the ways in which traditional craft cultures are produced, circulated, and interpreted. While prior research has examined digital heritage broadly, limited attention has been paid to how platform-based dissemination transforms ceramic culture in historically significant craft centers such as Jingdezhen.

**Methods:**

This study adopts a qualitative research design, combining semi-structured interviews with 32 ceramic practitioners and digital ethnography of 58 ceramic-related livestreaming sessions on Douyin.

**Results:**

The findings reveal three key dynamics: (1) the reconfiguration of craft authority through platform visibility; (2) the emergence of hybrid artisan–educator–entrepreneur identities; and (3) persistent tensions between cultural authenticity and commercial logic in platform-mediated environments.

**Discussion:**

By integrating cultural ecology and platform ecosystem theory, this study contributes to scholarship on digital heritage and provides practical insights for cultural practitioners and heritage institutions navigating digital platform ecosystems.

## 1 Introduction

The global rise of digital media and platform ecosystems has accelerated cultural dissemination and reshaped traditional cultural industries. Jingdezhen, known as the “Porcelain Capital” of China, presents a unique case for understanding how intangible cultural heritage adapts to the digital era. While existing scholarship has offered systematic analyses of digital heritage and platform-based cultural dissemination, the lived perspectives of cultural practitioners and audiences remain comparatively underexplored (Harrison, 2013; Smith and Akagawa, 2009). This study addresses this gap through qualitative inquiry grounded in interviews and ethnographic observation.

Recent empirical work on digital ceramic design, educational integration, and digital protection—such as studies on interactive narrative design of Jingdezhen ceramics (Cai and Tang, 2025), digital preservation (Gong, 2022; Fu, 2023; Li, 2025), and curriculum integration (Cui and Wang, 2021)—illustrates the increasing entanglement between heritage practices and digital ecosystems. UNESCO's operational directives for safeguarding intangible cultural heritage outline implementation procedures and emphasize community engagement and inclusive approaches to heritage transmission (UNESCO, 2018). Local initiatives in Jingdezhen, including digitization supported by universities and museums (Liu and Cheng, 2024; Wu, 2024; Cao and Ke, 2025), further demonstrate how cultural–digital intersectionality is being operationalized in practice.

## 2 Literature review

This section synthesizes scholarship on digital cultural dissemination, intangible cultural heritage digitization, and platform innovation relevant to Jingdezhen ceramic culture.

### 2.1 Digital dissemination of traditional culture

Studies increasingly recognize that digital technologies—such as livestreaming, short-video platforms, and VR/AR—are reshaping how traditional culture is represented and accessed. Digital media lower dissemination thresholds, diversify narrative forms, and enable personalized cultural consumption (Jin and Wu, 2024; Wang, 2023). Research on Chinese livestreaming and short-video ecosystems demonstrates that process-focused visual formats, such as wheel-throwing demonstrations or glazing processes, generate strong audience engagement and shape new consumption logics (Tang, 2022; Jin et al., 2025). Similar findings emerge in Jingdezhen-specific studies, where ceramic artisans use Douyin and Xiaohongshu to extend market reach and cultivate digital identities (Wang, 2024; Long, 2025). Recent research further highlights structural and tourism-related barriers influencing the digital transformation of Jingdezhen's art sector (Wang and Chen, 2024).

### 2.2 Digitization of intangible cultural heritage

ICH digitization research highlights both opportunities and risks. Digitization promotes preservation, education, and global reach (Yan et al., 2025; Meng and Zhang, 2025), but can also lead to cultural simplification, over-commercialization, and loss of embodied craftsmanship (Fu, 2023). UNESCO promotes community-led documentation, 3D/VR archiving, and multi-stakeholder participation to avoid reductive narratives (UNESCO, 2018). Case studies in Jingdezhen identify progress in digital archiving, integration into educational programs, and collaborative local initiatives (Liu and Cheng, 2024; Zhao et al., 2022; Cui and Wang, 2021). However, they also highlight challenges: balancing safeguarding with commercial platform pressures, ensuring authenticity, and dealing with uneven digital literacy (Li, 2025; Gong, 2022; Cao and Ke, 2025). Despite these advances, existing scholarship largely approaches digital dissemination, ICH digitization, and platform innovation as parallel or loosely connected processes. Much of the literature focuses either on technological affordances and representational outcomes, or on policy-oriented safeguarding frameworks, with limited attention to how artisans themselves navigate platform logics in their everyday cultural practice. In particular, the lived experiences of craft practitioners—how they negotiate authenticity, visibility, and economic survival within algorithmically governed environments—remain underexamined. Moreover, Jingdezhen is often treated as a case of successful digitization rather than as a dynamic cultural ecology shaped by ongoing interactions among platforms, institutions, and localized craft traditions. This study addresses these gaps by examining Jingdezhen ceramic culture as a hybrid cultural ecology, foregrounding artisans' perspectives and situating platform participation within localized cultural, economic, and institutional contexts.

### 2.3 Platform innovation and cultural industries

Platform studies emphasize the rise of algorithm-driven visibility, user co-creation, and data-mediated cultural production. Platforms serve as intermediaries shaping economic and symbolic value flows (Althaus, 2025). Research on Chinese platforms shows that business models, recommendation algorithms, and content-creation incentives shape which cultural practices gain visibility (Duan, 2024). ICH-related livestreaming studies indicate that algorithmic reward structures often privilege entertaining or sensationalized content, creating tensions between authenticity and algorithmic popularity (Yingqing, 2024; Zhang, 2025). In Jingdezhen's case, platform-mediated cultural production interacts closely with local artistic mobility and global cultural exchanges (Huang et al., 2025), suggesting that the ceramic ecosystem increasingly operates as a hybrid digital–physical cultural economy.

## 3 Theoretical framework

This study applies two complementary theoretical lenses that jointly explain how Jingdezhen ceramic culture adapts to digital transformation. A comparative overview of these two frameworks is provided in Table 1, which summarizes their core assumptions, analytical focus, and relevance to this study.

**Table 1:** Comparison of cultural ecology theory and platform ecosystem theory.

**Dimension**	**Cultural ecology theory**	**Platform ecosystem theory**
Primary focus	Interaction between culture and environment	Multi-sided interactions on digital platforms
Core assumptions	Culture adapts to ecological and institutional conditions	Digital platform environments structured by algorithms and governance shape participation
Unit of analysis	Cultural systems, communities, environments	Producers, intermediaries, users, and platform infrastructure
Mechanisms of change	Environmental adaptation, institutional evolution	Algorithmic visibility, monetization models, affordances
Relevance to Jingdezhen	Explains hybrid physical–digital craft ecosystems	Explains creator behavior, visibility, and digital cultural value
Key Scholars Referenced	de Bernard; Wu; Wei et al.	Duan; Althaus; Jin & Wu

### 3.1 Cultural ecology theory

Cultural ecology theory is particularly relevant to this study because it conceptualizes culture as an adaptive system shaped by ongoing interactions among material environments, social institutions, and external forces. Rather than treating digital platforms as neutral tools or external disruptors, cultural ecology allows this research to examine how platform technologies become embedded within localized craft environments and reorganize existing cultural relationships. Applied here, cultural ecology frames Jingdezhen ceramics as evolving within a hybrid physical–digital ecosystem comprising studios, local institutions, educational systems, and platform infrastructures (de Bernard, 2022; Wu, 2024; Cao and Ke, 2025). This perspective highlights how digital archives, platform economies, and university–industry collaborations do not operate in isolation, but interact with long-standing craft traditions, lineage-based knowledge transmission, and place-specific production networks.

By adopting a cultural ecology approach, this study is able to analyze not only technological change, but also processes of adaptation, negotiation, and resilience within craft communities. In Jingdezhen, digital platforms reshape transmission pathways, craft identities, and creative labor conditions, while remaining anchored in material practices and institutional structures (Cai and Tang, 2025; Liu and Cheng, 2024). Cultural ecology therefore provides a coherent theoretical framework for understanding how digital transformation unfolds as an ecological reconfiguration rather than a linear replacement of tradition.

### 3.2 Platform ecosystem theory

Platform ecosystem theory conceptualizes platforms as multi-sided structures involving producers, consumers, and intermediaries. As shown in Table 1, this framework highlights platform governance, data flows, and business archetypes as key forces shaping cultural outcomes (Duan, 2024; Althaus, 2025). Algorithms determine visibility; affordances shape participation; and monetization models influence creative behavior (Jin and Wu, 2024; Wang, 2024), providing critical insights into Jingdezhen's digital transformation.

### 3.3 Platform ecosystem theory

Platform ecosystem theory is employed in this study to analyze the digital platform environment in which cultural production and circulation are organized. Unlike cultural ecology theory, which emphasizes place-based cultural systems and institutional adaptation, platform ecosystem theory focuses on platform-mediated digital environments structured by algorithms, data flows, governance rules, and monetization mechanisms. Platform ecosystem theory conceptualizes platforms as multi-sided systems involving content producers, users, intermediaries, and platform operators, whose interactions are coordinated through technical architectures and economic incentives (Duan, 2024; Althaus, 2025). Within this digital platform environment, algorithms determine visibility, platform affordances shape participation formats, and monetization models influence creative behavior and professional strategies (Jin and Wu, 2024; Wang, 2024). Applied to the Jingdezhen context, this framework enables the analysis of how ceramic artisans and cultural creators navigate platform-specific logics—such as recommendation systems, engagement metrics, and livestreaming infrastructures—when presenting heritage knowledge online. Platform ecosystem theory thus provides critical insights into how cultural value, visibility, and labor are reorganized within digital environments that operate alongside, yet distinct from, physical craft spaces.

## 4 Methodology

### 4.1 Research design

This study adopts a qualitative, multiple-case research design to examine how Jingdezhen ceramic culture is reconfigured within platform-mediated digital environments. Given the complexity of contemporary cultural production shaped by algorithmic platforms, qualitative inquiry is well suited to capturing lived experiences, interpretive meaning-making, and multi-layered interactions among cultural actors. The research design integrates semi-structured interviews, digital ethnography, and field observation to enable triangulated analysis across both online and offline settings. This approach allows for an in-depth examination of how cultural production, mediation, and reception unfold within interconnected digital and physical contexts. Guided by platform ecosystem theory, the study adopts a multi-sided analytical perspective that emphasizes interdependence among producers, intermediaries, and users within algorithmically governed environments (Duan, 2024; Althaus, 2025). Accordingly, the multiple-case design encompasses three key stakeholder groups: (1) artisans and studio owners, (2) digital content creators, and (3) consumers and cultural tourists. This structure enables comparative analysis of how ceramic culture is produced, mediated, and interpreted across different positions within the platform ecosystem. An overview of the research design and stakeholder composition is presented in Table 2.

**Table 2:** Overview of the multiple-case research design.

**Stakeholder group**	**Sample size**	**Characteristics**	**Contribution to study**
Artisans & studio owners	12	Based in Taoxichuan and traditional kiln clusters	Provide insights into craft identity, production shifts, and digital adoption
Digital content creators	10	Active on Douyin, Xiaohongshu, Bilibili	Offer perspectives on algorithmic influence, persona creation, and platform strategies
Consumers & cultural tourists	10	Engage with Jingdezhen ceramics through digital media	Reveal audience expectations, learning behaviors, and cultural perception

Cases were selected to represent producer–intermediary–consumer dynamics consistent with platform ecosystem theory.

### 4.2 Study area

Jingdezhen is widely recognized as the “Porcelain Capital of China,” with a ceramic production history spanning over a millennium. It is a nationally recognized center of intangible cultural heritage, where traditional kiln systems, artisan lineages, and contemporary creative industries coexist. In recent years, Jingdezhen has also emerged as a prominent site of digital cultural experimentation, making it particularly suitable for examining the intersection of heritage practices and platform-based dissemination.

### 4.3 Participants and recruitment

A total of 32 participants were recruited through purposive sampling. Inclusion criteria required participants to have a minimum of three years of engagement in ceramic-related practice or sustained involvement in ceramic-related digital activities. Participants were selected to represent diverse roles within Jingdezhen's platform-mediated ceramic ecosystem, including artisans and studio owners (*n* = 12), digital content creators (*n* = 10), and consumers or cultural tourists (*n* = 10).

### 4.4 Data collection

#### 4.4.1 Semi-structured interviews

Data collection was conducted between March and September 2024 and involved 32 semi-structured interviews with key stakeholder groups. The sample comprised 12 artisans and studio owners, 10 digital creators—including livestreaming hosts, content educators, and craft vloggers—and 10 consumers or cultural tourists who follow, purchase, or learn ceramic knowledge through digital platforms. Interview questions focused on experiences with digital dissemination, perceptions of algorithmic dynamics, challenges related to authenticity and commercialization, opportunities for creative expansion, and interactions with digital archiving or intangible cultural heritage (ICH) systems. Each interview, lasting between 30 and 70 minutes, provided nuanced insights into how digital tools influence both professional identity and cultural value formation. The semi-structured format ensured consistency while allowing participants to elaborate on emergent themes relevant to evolving craft–platform relations. Interview distribution across stakeholder groups is summarized in Table 3, which illustrates the proportional composition of artisans, digital creators, and consumers included in the final sample.

**Table 3:** Summary of interview participants (N = 32).

**Participant group**	**Number**	**Percentage**	**Examples of roles**
Artisans/studio owners	12	37.5%	Ceramic artists, glaze technicians
Digital creators	10	31.25%	Livestreamers, vloggers, craft educators
Consumers/ tourists	10	31.25%	Hobbyists, collectors, cultural tourists

Interviews lasted 30–70 min.

#### 4.4.2 Digital ethnography

Digital ethnography was employed to examine how ceramic-related cultural practices are produced, circulated, and interpreted within platform-mediated environments. The ethnographic focus was placed on social media platforms that play a central role in China's contemporary cultural and creative economy, specifically Douyin, Xiaohongshu, and Bilibili. These platforms were selected due to their distinct affordances for livestream-based commerce, visual curation, and educational or process-oriented content related to ceramics. Between March and September 2024, the researcher systematically observed 58 ceramic-related livestreaming sessions on Douyin. These livestreams were primarily hosted by ceramic artisans, studio operators, or affiliated content creators based in Jingdezhen. Observations focused on modes of creator performance, demonstrations of ceramic techniques, real-time commercial interactions, and the ways in which cultural narratives were articulated through live engagement with audiences. Particular attention was paid to how streamers balanced craft explanation, product promotion, and audience interaction within algorithmically structured environments.

In addition to livestreaming sessions, a corpus of 132 short-form videos was collected from Xiaohongshu and Bilibili, including 76 videos from Xiaohongshu and 56 from Bilibili. These videos were selected based on their explicit focus on Jingdezhen ceramics, craft processes, studio life, or ceramic-related cultural storytelling. Observational documentation included visual aesthetics, narrative framing, identity presentation, and viewer engagement patterns such as comments, reposts, and expressions of learning or affective response. Ethnographic data collection also involved the documentation of audience interactions and comment threads associated with both livestreams and short videos. Over 3,400 comments were reviewed to identify recurring patterns of audience participation, including questions about technique, expressions of admiration, purchasing intentions, and forms of emotional or symbolic support. In addition, algorithmic recommendation trajectories were recorded during browsing sessions to capture platform-generated cues that influenced content visibility and user navigation.

References to digital–physical integrations, such as QR-code catalogs, AR-enhanced exhibits, and digital heritage interfaces encountered during field visits or mentioned by participants, were treated as contextual observations rather than core methodological components. These elements were documented to situate platform-based practices within a broader hybrid cultural environment but were not analyzed as independent technological interventions.

All ethnographic observations were recorded through structured fieldnotes and integrated with interview data during the analytical process. Rather than aiming for representativeness or frequency-based generalization, the digital ethnography sought to capture recurring practices, interactional patterns, and meaning-making processes that characterize platform-mediated ceramic culture in Jingdezhen. An overview of the digital ethnography dataset, including platform distribution, data types, quantities, and observational emphases, is provided in Table 4.

**Table 4:** Digital ethnography dataset overview.

**Platform**	**Data type**	**Quantity**	**Observational focus**
Douyin	Livestream sessions	58	Creator performance, commerce interaction
Xiaohongshu	Short videos	76	Aesthetic curation, identity display
Bilibili	Short videos	56	Educational content, process demonstrations
All	Comment threads	3,420+	Audience reactions, fan labor, emotional participation

Algorithm recommendation paths were recorded during browsing sessions.

#### 4.4.3 Field observation

Two field visits to Jingdezhen (April and July 2024) provided contextual grounding. During these visits, the researcher conducted participant observation inside ceramic studios, visited the Taoxichuan Art District, and toured museums and digital heritage exhibitions. Particular attention was paid to digital–physical integrations such as QR-code catalogs, AR-enhanced exhibits, digital retail interfaces, and creator incubation spaces. Fieldnotes documented spatial interactions, artisan–visitor exchanges, and the hybridization of local heritage spaces with digital infrastructure. These observations illuminated how Jingdezhen constitutes an evolving cultural ecology in which physical craft environments coexist with digital circulation systems (Wu, 2024; Cao and Ke, 2025). The integration of field observation data enhances the ecological validity of the qualitative findings. Key field sites and their corresponding digital–physical integration features are outlined in Table 5, which details the role of museums, studios, and cultural districts in Jingdezhen's hybrid creative ecology.

**Table 5:** Field observation activities in Jingdezhen (2024).

**Location/activity**	**Description**	**Purpose**
Taoxichuan art district	Studio visits; creator incubator observation	Examine digital–physical integration
Traditional kiln clusters	Process observation; artisan interaction	Understand traditional craft ecology
Museums/AR–VR exhibits	Digital heritage systems	Document hybrid heritage display formats
University collaborations	Digitization and AI heritage labs	Explore institutional digital innovation

### 4.5 Data analysis

All interviews were audio-recorded and transcribed verbatim. The complete dataset—including transcripts, fieldnotes, and digital ethnography materials—was analyzed using thematic analysis following Braun and Clarke's (2006) three-phase approach: initial coding, axial category development, and theme construction. Coding combined deductive strategies guided by cultural ecology and platform ecosystem theory with inductive processes that captured emergent concepts from participant narratives. Notably, inductive categories such as “algorithmic fatigue,” “persona-driven craft identity,” and “digitally enhanced authenticity” surfaced during analysis. Cross-case comparison enabled the identification of convergent and divergent patterns across artisans, digital creators, and consumers. This analytic process ensured a robust interpretation of how craft practices, platform logics, and cultural values interact in contemporary Jingdezhen. The three-phase coding procedure and theme-development process follow Braun and Clarke's framework, with the coding structure summarized in Table 6, illustrating how initial codes were consolidated into broader analytical themes. Coding was conducted iteratively, with constant comparison across stakeholder groups. To enhance analytical rigor, themes were reviewed multiple times against the original data to ensure internal consistency and theoretical coherence.

**Table 6:** Thematic analysis coding phases.

**Phase**	**Description**	**Output**
Initial coding	Line-by-line and node-based coding	215 initial codes
Axial categorization	Clustering and relational mapping	19 axial categories
Theme development	Higher-level pattern identification	4 major themes
Cross-case comparison	Triangulation across groups	Convergent/divergent patterns

### 4.6 Researcher reflexivity

Given the qualitative and interpretive nature of this study, researcher reflexivity was systematically incorporated throughout the research process. The researcher maintained reflexive fieldnotes during interviews, digital ethnography, and on-site observations to critically examine how positionality, assumptions, and interactions with participants might influence data collection and interpretation. Prolonged engagement in Jingdezhen facilitated rapport-building with artisans, digital creators, and institutional actors, enabling access to both formal interviews and informal contextual insights. At the same time, reflexive practices were employed to mitigate the risk of over-identification with participants' narratives, particularly in relation to discourses of cultural preservation, authenticity, and digital innovation. Analytic memos were used during coding to distinguish between participants' emic perspectives and the researcher's theoretical interpretations.

Reflexivity was further supported through iterative comparison across data sources, including interviews, digital ethnography, and field observations. This process helped ensure that analytical claims were grounded in empirical material rather than shaped by preconceived expectations about digital platforms or heritage transformation. By foregrounding reflexive awareness, the study enhances the credibility and interpretive rigor of its qualitative findings.

### 4.7 Ethical considerations

Ethical approval for this study was obtained from the relevant institutional ethics committee prior to data collection. All interview participants were informed of the study's purpose, procedures, and intended academic use of the data, and written informed consent was obtained before participation. Participants were assured of their right to withdraw at any stage without consequence. To protect privacy and confidentiality, all interview data were anonymized through the use of pseudonyms, and identifying details related to individuals, studios, or commercial operations were removed from transcripts and publications. Audio recordings and transcripts were securely stored and accessible only to the research team.

Digital ethnography was conducted using publicly accessible content from social media platforms, including livestreams, short videos, and comment threads. In accordance with ethical guidelines for internet research, platform-based observations focused on publicly available materials and avoided intrusive interaction. Where creators' content was referenced analytically, care was taken to anonymize accounts and avoid direct attribution unless explicit permission was granted. These measures ensure that the study adheres to ethical standards of research integrity, participant protection, and responsible use of digital data.

## 5 Findings and discussion

### 5.1 Findings

#### 5.1.1 Platforms as new creative and economic infrastructures

Findings indicate that digital platforms have become central infrastructures for artisans and studios in Jingdezhen. Participants consistently reported that Douyin, Xiaohongshu, and Taobao Live function as primary channels for visibility, customer acquisition, and product sales. Many artisans described platform engagement as a prerequisite for maintaining studio operations and reaching audiences beyond local markets. Participants highlighted several perceived advantages of platform use. These included lower entry barriers for younger practitioners, flexible forms of micro-entrepreneurship, and access to niche consumer communities unconstrained by geographical location. For some interviewees, digital dissemination was described as essential for sustaining their practice amid increasing competition within Jingdezhen's craft economy. Patterns of platform usage reported by artisans and creators—including dominant functions, perceived opportunities, and experienced pressures—are summarized in Table 7. These patterns illustrate how different platforms are used for commercial activity, visibility-building, educational communication, and audience engagement.

**Table 7:** Artisan-reported platform dependence (code frequency).

**Platform function**	**Frequency of mentions**	**example meaning**
Sales/commerce	48	Taobao Live as primary revenue channel
Visibility/branding	42	Douyin exposure increasing follower base
Teaching/education	26	Explaining processes via short videos
Technological pressure	18	Need to adapt to algorithms

Frequencies represent coded segment counts.

#### 5.1.2 Reconfigured craft identity and digital personas

A second recurring theme concerns shifts in craft identity associated with sustained platform participation. Participants described adopting hybrid roles that combine traditional craftsmanship with content creation, education, and online self-presentation. Commonly cited identity labels included “artisan–educator,” “craftsperson–performer,” and “artisan–entrepreneur.”While some participants expressed enthusiasm about these expanded roles, others reported ambivalence or discomfort. Several artisans noted pressure to continuously perform, narrate, or aestheticize their work in ways that differed from established workshop-based practices. These identity shifts were frequently discussed in relation to audience expectations, platform visibility metrics, and perceived norms of successful content creation. The types of digital craft personas identified through participant narratives are summarized in Table 8, which outlines key characteristics and associated platform behaviors.

**Table 8:** Types of digital craft personas.

**Persona type**	**Characteristics**	**Associated platform behavior**
Artisan–educator	Demonstrates processes; tutorials	Step-by-step videos
Artisan–performer	Emphasizes entertainment	Livestream humor, fan interaction
Artisan–entrepreneur	Focus on sales, branding	Product demos, discount campaigns
Artisan–aesthetic curator	Visual storytelling	Studio aesthetics, lifestyle imagery

#### 5.1.3 Algorithmic mediation and authenticity tensions

Participants frequently reported tensions between established craft practices and platform-driven content dynamics. Short-video platforms were described as favoring visually striking, fast-paced, and easily consumable representations of ceramic production. In contrast, slow and time-intensive techniques—often regarded by artisans as central to Jingdezhen's craft tradition—were perceived as receiving limited visibility. Livestreaming practices were similarly described as encouraging performative interaction, emotional engagement, and entertainment-oriented presentation. Some participants noted that these dynamics could overshadow detailed technical explanation or historical contextualization of ceramic work.

Observational data from digital ethnography further indicate that platform algorithms shape content visibility in ways that influence how ceramic knowledge and cultural value are presented. Key platform-specific preferences and constraints identified through interviews and observation are consolidated in Table 9.

**Table 9:** Platform-specific algorithmic preferences (derived from observation).

**Platform**	**Favored content**	**Disfavored content**
Douyin	Fast-paced, high-sensory craft clips	Slow, contemplative processes
Xiaohongshu	Aesthetic, lifestyle-oriented visuals	Technical explanations
Bilibili	Long-form educational content	Commerce-heavy videos

#### 5.1.4 Hybrid cultural ecology in Jingdezhen

Field observations reveal that ceramic production and cultural dissemination in Jingdezhen increasingly operate across interconnected physical and digital spaces. Studios frequently integrate QR-code catalogs linking physical products to online shops or livestream sessions. Museums and exhibition spaces incorporate digital interfaces, including AR- and VR-supported displays, to supplement heritage interpretation. In addition, creator incubation spaces in areas such as Taoxichuan support the convergence of artisanal practice and digital entrepreneurship.

University–industry collaborations observed during fieldwork emphasize digitization processes, experimental multimedia documentation, and emerging digital tools for cultural transmission. Together, these observations illustrate the presence of a hybrid environment in which physical heritage sites, institutional actors, and digital platforms intersect. The structural components of this hybrid cultural–digital ecology are outlined in Table 10.

**Table 10:** Components of Jingdezhen's hybrid cultural–digital ecology.

**Domain**	**Digital component**	**Physical component**	**Interaction**
Retail	Online shops, livestream commerce	Studios and stores	QR-linked product lines
Education	Digital tutorials, VR tools	Universities, workshops	Hybrid teaching
Heritage	3D archives, AR exhibits	Museums, kiln sites	Digital-enhanced interpretation

### 5.2 Discussion

#### 5.2.1 Reinterpreting craft in the platform era

Building on the empirical findings, this study suggests that participation in digital platforms has contributed to a reinterpretation of what it means to practice craft in contemporary Jingdezhen. Artisans increasingly negotiate professional identities that extend beyond production alone, incorporating elements of education, performance, and entrepreneurial self-presentation. This pattern reflects broader transformations observed in digital cultural industries, where creators adopt multifaceted roles in response to platform-based modes of visibility and engagement (Jin and Wu, 2024).

Within the context of Jingdezhen, such role hybridity expands creative and economic opportunities, particularly for younger practitioners and small studios. At the same time, it reshapes hierarchies of cultural value by favoring practices that align with platform logics—such as visual immediacy, narrative accessibility, and audience interaction—over slower, process-intensive techniques traditionally associated with craft mastery. Rather than signaling a uniform decline or enhancement of craftsmanship, these shifts point to a redefinition of artisanal legitimacy under conditions of digital mediation. Audience expectations, in turn, are recalibrated as craft is increasingly encountered through platform-specific formats rather than workshop-based or institutional settings.

#### 5.2.2 Algorithmic governance as cultural governance

The findings further indicate that algorithmic visibility operates as an implicit form of cultural governance within platform-mediated environments. Through mechanisms of ranking, recommendation, and engagement-based amplification, platforms influence which forms of ceramic knowledge, aesthetics, and narratives gain prominence. While these systems are designed primarily to optimize user attention and platform activity, they simultaneously shape cultural meaning by privileging certain representations over others.

This study extends existing discussions in platform governance literature by foregrounding the cultural implications of algorithmic mediation (Althaus, 2025). Similar concerns regarding algorithm-driven simplification of intangible cultural heritage have also been noted in recent studies (Yingqing, 2024; Zhang, 2025). In the domain of intangible cultural heritage, algorithmic governance introduces structural tensions between marketplace-driven visibility and the preservation of contextual depth, technical complexity, and historical continuity. Table 11 synthesizes these contrasts by comparing traditional forms of cultural governance with emerging algorithmic logics, highlighting shifts in decision-making authority, evaluation criteria, and modes of knowledge transmission. Rather than suggesting a simple replacement of institutional governance, the findings point to a layered governance environment in which algorithmic systems coexist with museums, heritage authorities, and educational institutions. This coexistence raises critical questions about how authenticity, authority, and cultural responsibility are negotiated when heritage dissemination increasingly depends on opaque and data-driven infrastructures.

**Table 11:** Comparison of traditional cultural governance and algorithmic cultural governance.

**Dimension**	**Traditional cultural governance**	**Algorithmic cultural governance**
Core decision-maker	Cultural institutions, heritage authorities, experts	Platform algorithms, recommendation systems, engagement-based ranking
Cultural value logic	Emphasis on authenticity, lineage, historical continuity	Emphasis on attention, engagement, virality, speed
Content evaluation criteria	Expert review, cultural significance, craft quality	Viewer retention, click-through rate, interaction metrics
Visibility mechanism	Curatorial selection, institutional endorsement	Algorithmic amplification, personalized feeds
Gatekeeping actors	Museums, academies, cultural committees	Platform operators, algorithm engineers, opaque system rules
Representation of heritage	Slow, process-oriented, context-rich	Fast-paced, visually stimulating, simplified demonstrations
Knowledge transmission mode	Formal education, apprenticeships, museum interpretation	Short videos, livestreaming, influencers, user-generated content
Cultural risks	Institutional rigidity, limited audience reach	Popularity bias, over-commercialization, loss of depth and authenticity
Opportunities	Preservation accuracy, expert-led safeguarding	Broad dissemination, real-time interaction, youth engagement
Power dynamics	Top-down, expert-centered	Data-driven, platform-centered, creator-dependent

#### 5.2.3 Hybrid cultural ecology and localization

Finally, the case of Jingdezhen demonstrates that digital platforms are embedded within a localized cultural ecology rather than operating as external or homogenizing forces. The findings indicate that digital technologies do not replace physical craft environments but instead amplify, reorganize, and extend them. Studios, museums, universities, and creative districts form interconnected networks in which digital tools support documentation, experimentation, education, and global outreach, while remaining anchored in place-based ceramic traditions.

This hybrid configuration aligns closely with cultural ecology theory, which emphasizes adaptation, interdependence, and environmental responsiveness in cultural systems (de Bernard, 2022; Wu, 2024; Cai and Tang, 2025; Cao and Ke, 2025). In Jingdezhen, digital platforms facilitate new forms of collaboration and mobility—such as artist residencies, maker programs, and translocal exchanges—while continuing to rely on the material infrastructures, social relations, and symbolic authority of the local craft environment. Digital engagement thus operates not as a deterritorializing force, but as a mechanism through which locality is rearticulated and made legible to wider audiences.

Importantly, this localized hybrid ecology contributes to cultural resilience by enabling traditional practices to engage with global markets and publics without severing their material, historical, and social foundations. At the same time, the Jingdezhen case underscores that such outcomes are not automatic. As noted by (Wu 2024) and (Cai and Tang 2025), the sustainability of digitally mediated cultural ecosystems depends on institutional coordination, contextual sensitivity, and governance frameworks that balance technological innovation with cultural safeguarding. Localization therefore emerges as a critical condition through which digital platforms can support, rather than undermine, the long-term vitality of intangible cultural heritage.

## 6 Conclusion

This study examined how Jingdezhen ceramic culture is reconfigured within platform-mediated environments, drawing on qualitative interviews, digital ethnography, and field observation. The findings demonstrate that digital platforms and media ecosystems are profoundly reshaping the production, dissemination, and perception of ceramic culture, positioning platforms as integral infrastructures for cultural production, economic survival, and symbolic representation in contemporary craft ecosystems.

The study reveals that platform participation reshapes craft practices in three interconnected ways. First, digital platforms function as creative and economic infrastructures that lower entry barriers for artisans while simultaneously generating new dependencies on algorithmic visibility. Second, artisans and digital creators increasingly construct hybrid professional identities that blend craftsmanship, pedagogy, and performance, redefining what it means to be a craft practitioner in the platform era. Third, algorithmic affordances and reward structures act as implicit forms of cultural governance, selectively amplifying certain representations of heritage while marginalizing slower, less performative practices central to traditional craft knowledge, thereby generating ongoing negotiations around authenticity.

By situating these dynamics within a localized cultural ecology, the study contributes to broader debates in cultural heritage scholarship and platform studies. Rather than homogenizing local culture, digital platforms in Jingdezhen operate as mediating environments that reorganize relationships among physical practices, digital technologies, and institutional collaborations. This hybrid cultural–digital ecosystem enables local traditions to engage global audiences while remaining anchored in place-based material and social contexts, enhancing cultural resilience but also introducing new governance challenges.

The study offers several implications. Theoretically, it extends platform ecosystem theory into the domain of intangible cultural heritage, highlighting the cultural consequences of algorithmic mediation beyond economic or informational domains. Empirically, it provides a grounded account of how digital transformation is negotiated within a historically embedded craft center. Practically, the findings suggest that sustainable digital heritage development requires policy frameworks that balance platform participation with safeguarding concerns, including platform governance guidelines, community-led digital archiving, and artisan training programs that integrate digital literacy with heritage preservation.

Several limitations of this study warrant consideration. The study focuses on a single cultural site, and future research could pursue cross-regional comparisons or mixed-methods approaches to examine how algorithmic systems shape cultural visibility across different platform regimes. Longitudinal research may further illuminate how evolving platform logics influence craft practices over time. Nonetheless, this research demonstrates that the future of craft heritage is neither purely traditional nor fully digital, but unfolds within hybrid cultural ecologies in which technology, place, and practice continuously co-evolve.

## Data Availability

The original contributions presented in the study are included in the article/supplementary material, further inquiries can be directed to the corresponding author.
